# Utilizing Network Pharmacology to Explore the Possible Mechanism of Coptidis Rhizoma in Kawasaki Disease

**DOI:** 10.3389/fped.2021.708553

**Published:** 2021-09-13

**Authors:** Xue Fan, Xin Guo, Ying Li, Mingguo Xu

**Affiliations:** ^1^Department of Pediatric Cardiology, Shenzhen Children's Hospital, China Medical University, Shenzhen, China; ^2^Department of Pediatric, Longgang District Maternal and Children Health Care Hospital, Shenzhen, China

**Keywords:** Kawasaki disease, network pharmacology, Coptidis Rhizoma, Western blotting, GO and KEGG

## Abstract

**Background:** The purpose of the research is to identify the main active ingredients in Coptidis Rhizoma (CR) and explore the possible molecular mechanisms in the treatment of Kawasaki disease (KD).

**Materials and Methods:** A total of 58 children with KD were randomly divided into a control group and a Berberine treatment group. The therapeutic indicators of the two groups before and after treatment were compared. Then, compounds and drug targets of CR from the TCMSP, SWISS, SEA, and the STITCH were collected, and targeted KD genes were retrieved from the DisGeNET, DrugBank, and GeneCards databases. The network pharmacology approach involved network construction, target prediction, and module analysis. GO and KEGG enrichment analysis were performed to investigate the possible pathways related to CR for KD treatments. Finally, protein expression was determined to verify the core targets using Western blotting in the cell experiment.

**Results:** In total, nine compounds, 369 relative drug targets, and 624 KD target genes were collected in the above database. The network analysis revealed that 41 targets might be the therapeutic targets of CR on KD. GO and KEGG enrichment analysis revealed that the biological processes, namely, response to hormone, response to inorganic substance, and enzyme-linked receptor protein signaling pathway, and Pathways in cancer, Toll-like receptor signaling pathway, and Pancreatic cancer are the most significant. Protein expression of CASP3, PTGS2, and SRC was upregulated and AKT1 and ERK were downregulated.

**Conclusion:** We provided useful resources to understand the molecular mechanism and the potential targets for novel therapy of KD.

## Introduction

Kawasaki disease (KD) is a systemic inflammatory vasculitis predominantly affecting children younger than 5 years of age (1) and is now the most common acquired heart disease among children in North America, Europe, and Japan (2). KD can cause permanent vascular complications, especially coronary artery aneurysms (CAA) that may result in myocardial infarction (3, 4) and may also be associated with serious cardiovascular sequelae in adulthood (5). The standard treatment for KD includes intravenous gamma globulin (IVIG) and high-dose aspirin. However, up to 15–20% of patients with KD do not respond to IVIG treatment and have an increased rate of CAA (6). Yet, the underlying mechanisms of KD remain unclear and it may be related to unbalanced autoimmunity and genetic susceptibility (7). It is important to systematically elucidate the mechanism of the disease and search for more effective agents against it.

Coptidis Rhizoma (CR), called Huang Lian (HL) in Chinese, belongs to perennial herbs of *Ranunculaceae*. The Isoquinoline Alkaloids in Coptis Chinensis, namely Berberine (BBR), Coptis Chinensis, and Rhizopine, are the main effective components of CR. Data have proved that CR has anti-inflammatory (8), antibacterial (9), antitumor (10), antidiabetics (11), and pharmacological effects on the cardiovascular system, mainly related to the effects of BBR. A previous study in the same field has reported that BBR protected endothelial progenitor cells from injury induced by tumor necrosis factor α *via* the Phosphatidyl Inositol 3-kinase (PI3K)/serine/threonine protein kinase B/endothelial nitric oxide synthase signaling pathway (12). In addition, another examination confirmed that BBR protects KD-induced human coronary artery endothelial cells dysfunction by inhibiting oxidative and endoplasmic reticulum stress (13).

Network pharmacology is a part of bioinformatics based on the concept of “disease–gene–target–drug” (14), which is a new and effective means to study the mechanisms of drug therapy (15). Using network pharmacology, the relationship between drugs and diseases is analyzed from the molecular level, multi-target, and multi-pathway, potentially providing useful information in the search for drug treatment. Finally, we used Western blotting to measure the protein levels of the hub proteins.

To investigate the clinical effects of BBR on patients with KD and its pharmacological mechanisms, a randomized and case–control study was performed in Shenzhen Children's Hospital and a comprehensive network pharmacology approach was established to probe the appliances of CR on KD by network pharmacology analysis.

## Materials and Methods

### Human Serum Samples

The sample group of this study is formed by 20 children with KD (average age:1.80 years; ratio of male/female: 11/9) admitted to the Department of Cardiology and 20 healthy children (average age:1.90 years; ratio of male/female: 11/9) from Child Health Section, Shenzhen Children's Hospital from October 2018 to May 2019. They have randomly divided them into the routine treatment group and the routine treatment + BBR group. All children were typical KD patients and the diagnostic criteria were in line with the AHA and American Academy of Pediatrics 2004 guidelines (16). The KD serum samples were collected on the day the patients were hospitalized before IVIG therapy. In addition, these patients did not have CALs and were not IVIG resistant. The healthy children were collected randomly in the Child Health Section at the same stage. After being divided, the kids were treated with routine treatment or the combination of routine treatment and BBR (10 mg/kg, per time, bid) orally. Three days after treatment, hematologic examination and IVIG resistance rate were compared between the two groups. The research protocol was approved by the Ethics Committee of Shenzhen Children's Hospital (NO. 20180601). Serum samples were separated by centrifugation at 1,000 × *g* for 10 min and aliquots were stored at −80°C.

### Collection of Chemical Components in CR

The chemical components were all collected from Traditional Chinese Medicine Systems Pharmacology (TCMSP) Database (http://lsp.nwu.edu.cn/tcmsp.php) (17), a system pharmacology platform designed for studying TCMs comprehensively. In this process, drug-likeness (DL) values ≥0.18, oral bioavailability (OB) ≥30% and blood-brain barrier (BBB) ≥ −0.3 were selected as the active ingredients for the next step (18). In addition, the compounds with high contents and significant pharmacological activities that did not meet the requirements were also retained.

### KD Target Database Building

Information on KD-associated target genes was collected from the following resources, including Drugbank Database (https://www.drugbank.ca/) (19), GeneCards Database (https://www.genecards.org/) (20) and DisGeNET Database (http://www.disgenet.org/home/) (21). It is noteworthy that the repetitive genes collected from the mentioned sources were removed.

### Target Fishing for CR

The active ingredients of drugs play an important role in related biological functions *via* targets. The target fishing was used to search for or predict the potential targets of small molecules and the small molecular structure information of the active ingredients in CR by TCMS could also be predicted by applying the Similarity Ensemble Approach (22) (SEA, http://sea.bkslab.org/), STITCH (23) (http://stitch.embl.de/), and Swiss Target Prediction (24) (Swiss, http://www.swisstargetprediction.ch/).

### Construction and Analysis of the Pharmacological Networks

In this phase, the CR targets and the acquired KD targets were screened by the online tools Venny2.1.0 (http://bioinfogp.cnb.csic.es/tools/venny/index.html) to draw the Venny map and also to obtain the common target genes of the intersection. Then, the online tool STRING (http://string-db.org) was used to construct the target protein–protein interaction (PPI) network of CR acting on KD. The network construction was established, first, with a network between active compounds and targets of CR and also with PPI network of compounds and targets developed by linking the compound targets and predicted targets of other human proteins. Another PPI network of KD targets was also constructed by linking the known KD-related target.

### Gene Ontology (GO) and Pathway Enrichment Analysis

To investigate the functional annotation and involved pathways of genes, we used the Gene Ontology (GO) and the Kyoto Encyclopedia of Genes and Genomes (KEGG) enriched by GSEA (25) with FDR <0.001.

### Human Subjects and Serum Sample Collection

In our cell experiment, a total of 28 subjects, including 14 normal children and 14 KD patients, were recruited from Shenzhen Children's Hospital. Serum samples were separated by centrifugation at 1,000 × *g* for 10 min and aliquots were stored at −80°C.

### HCAECs Culture and Treatment

HCAECs were brought form ScienCell (San Diego, CA, USA) and cultured with endothelial cell growth medium containing growth factors, supplements, and 10% fetal bovine serum at 37°C in 5%CO_2_. Cells were then stimulated with culture medium containing 15% serum [15% (V/V)] from KD patients or healthy donors for 24 h. The cells were then used in subsequent studies. Each measurement was performed in triplicate (*n* = 3). The BBR was dissolved in sterile ultrapure water. The stock solution of BBR was 40 μm, which can be diluted into final concentration (20 μm) with cell culture medium.

### Western Blotting Analysis

The protein samples of the three groups were extracted and measured through a BCA protein assay. We loaded and separated the proteins through 4 and 10% SDS-PAGE and then transferred them onto PVDF membranes. The membranes were washed and then blocked using 5% skimmed milk powder blocking solution for 2 h. The antibody (Anti-β-actin, Anti-PTGS2, Anti-AKT1, Anti-CASP3, Anti-ERK, and Anti-SRC) was diluted at 1:1,000, diluted with 5%BSA TBST, and incubated overnight at 4°C. After washing three times with TBST for 4 min each, the membranes were incubated with secondary antibodies (Goat Anti-Mouse IgG, Goat Anti-Rabbit IgG) for 2 h at room temperature. Then, the membranes were washed four times with TBST for 4 min each. Finally, the image is collected and analyzed.

### Statistical Analysis

Here, all quantitative data are presented as means ± SEM. For pairwise comparison of two or more groups of quantitative data, *p*-values were calculated by *t*-test and chi-square, as appropriate. *p* < 0.05 was regarded as having statistical significance.

## Results

### Clinical Research Outcomes

**Figure 1:**
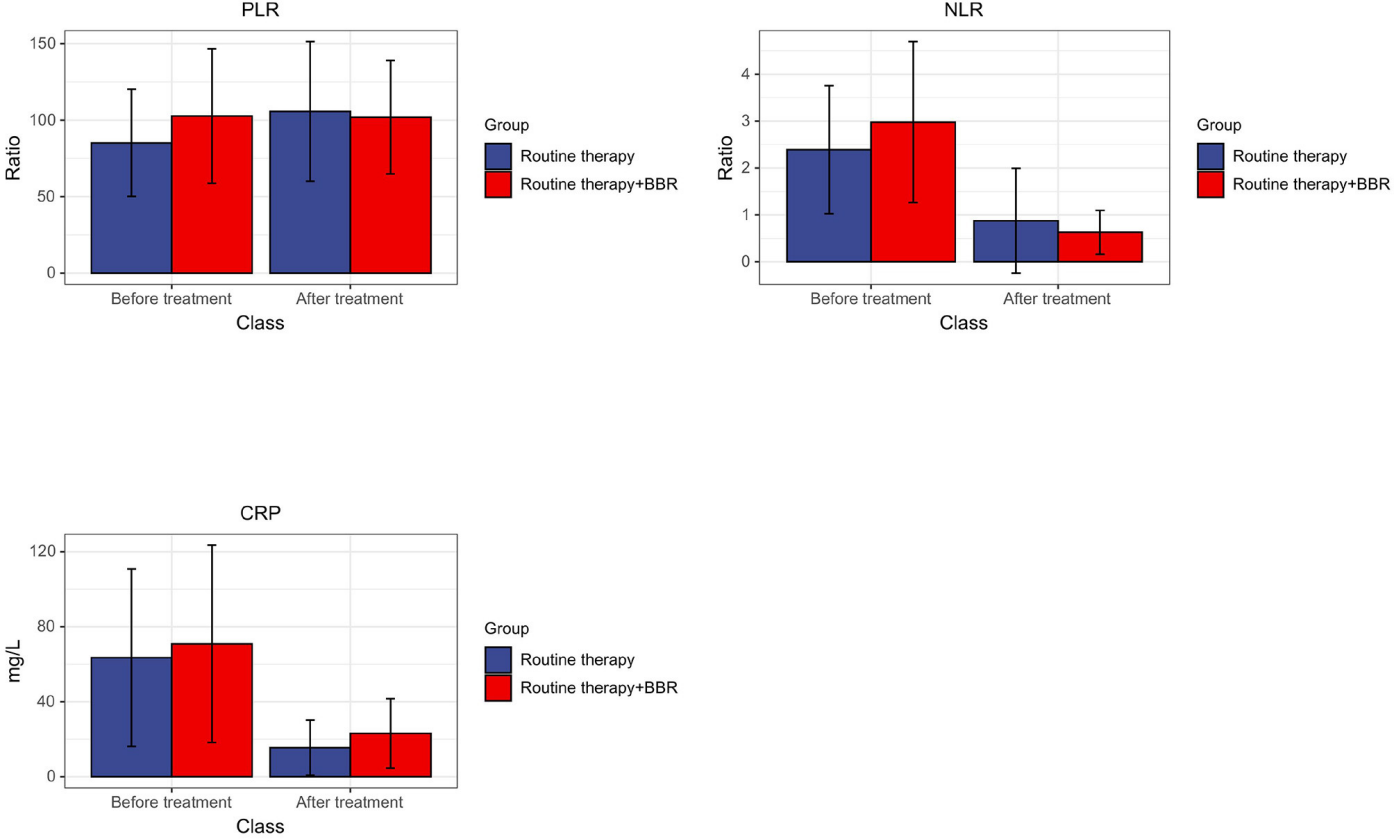
Changes in the average value of CRP, NLR, and PLR before and after treatment between the routine group and the combined group.

One of the first things to be observed in this phase is that no statistically significant differences were found in baseline data and hematologic indicators between the two groups. The BBR group was able to reduce the values of C-reactive protein (CRP), neutrophils/lymphocytes (NLR), and platelets/lymphocytes (PLR) (shown in Figure 1), and there was no statistical divergence in the number of days with fever, WBC, PLT, AST, ALT, and other blood indexes between the samples. Also, the resistance rate of IVIG decreased after in the BBR group.

### Active Compounds in CR and Target Screening of KD

**Figure 2:**
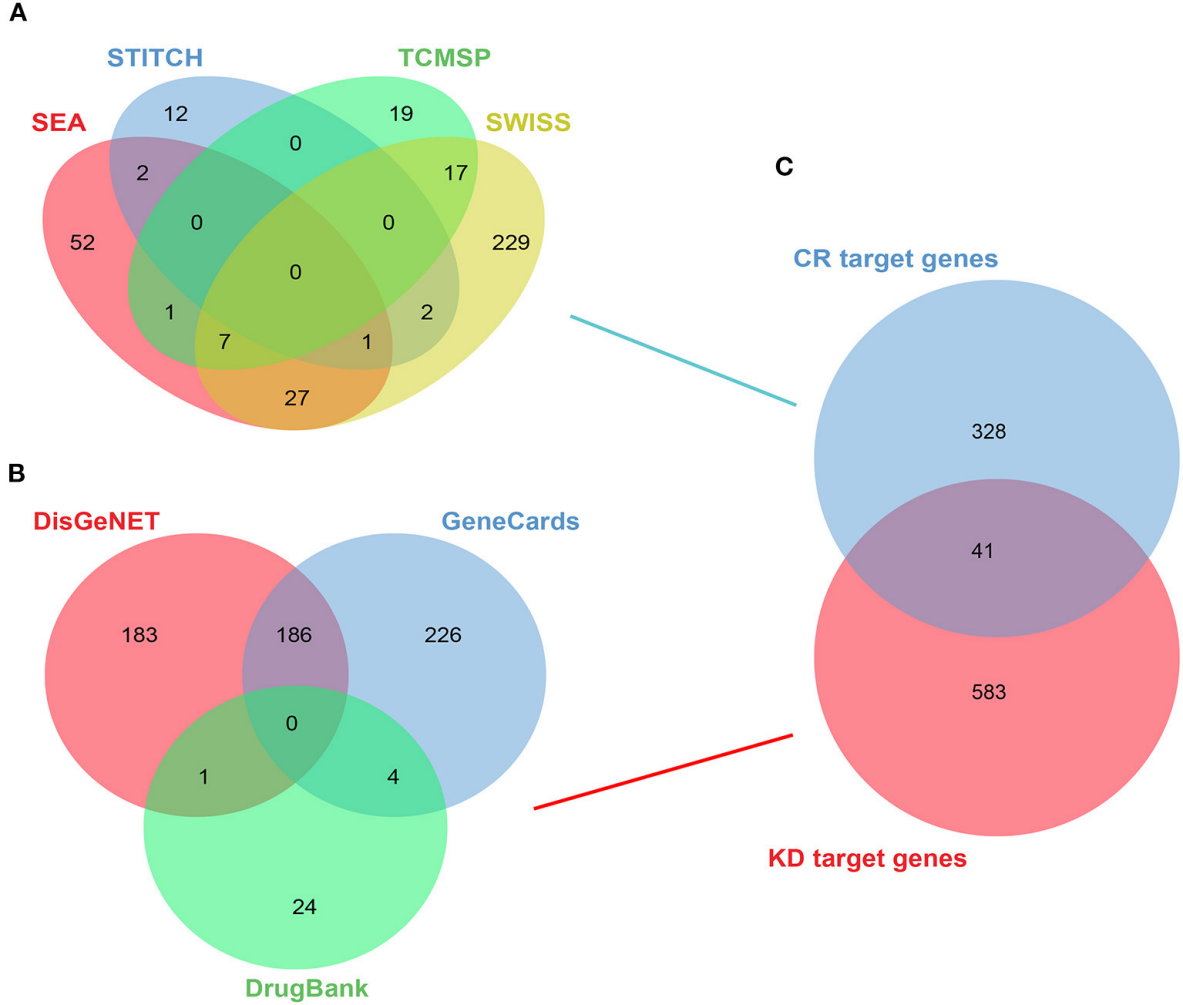
Active compounds in CR, target screening of KD, and the intersection between them. **(A)** Target genes of CR from TCMSP, SWISS, SEA, and STITCH databases. **(B)** Target genes of KD from DisGeNET, DrugBank, and GeneCards databases. **(C)** Venn diagram of the intersection of CR gene and KD gene.

Overall, nine target compounds were collected from TCMSP and 369 target genes in the whole formula in total were retrieved from TCMSP, SWISS, SEA, and STITCH database (Figure 2A). The values of OB, DL, and BBB were used to screen potential active compounds and the detailed properties of the compounds are shown in Supplementary Table S1. A total of 624 significant genes were gathered from the DisGeNET, DrugBank, and GeneCards databases after deleting repetitions (Figure 2B). KD was used as a keyword for retrieval, screening, and sorting out related targets and the Venn diagram was applied to show the intersection of disease targets and component targets. Finally, 41 therapeutic target genes of CR for KD could be obtained (Figure 2C).

### Compound–Compound Target Network Analysis

**Figure 3:**
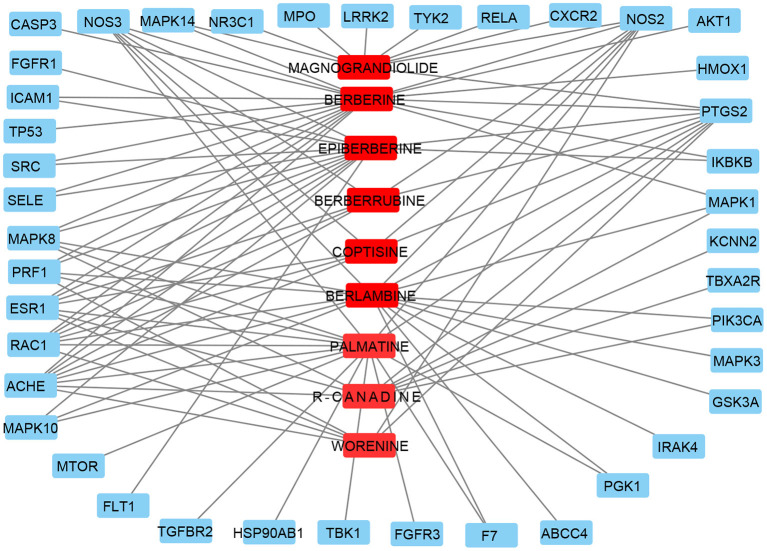
Compound–target genes network. Red circles represent active ingredients in CR. Blue rectangles represent targets of CR. Edges represent interaction between ingredients and targets.

To further determine the target of CR on KD, a compound-target network was built with an online tool Cytoscape3.6, representing a compound-target gene network including nine compounds and 41 target genes (shown in Figure 3). Compound BERBERINE has most target genes, 18, and only one target gene PTGS2 has all nine compounds.

### GO and KEGG Pathway Enrichment Analysis

**Figure 4:**
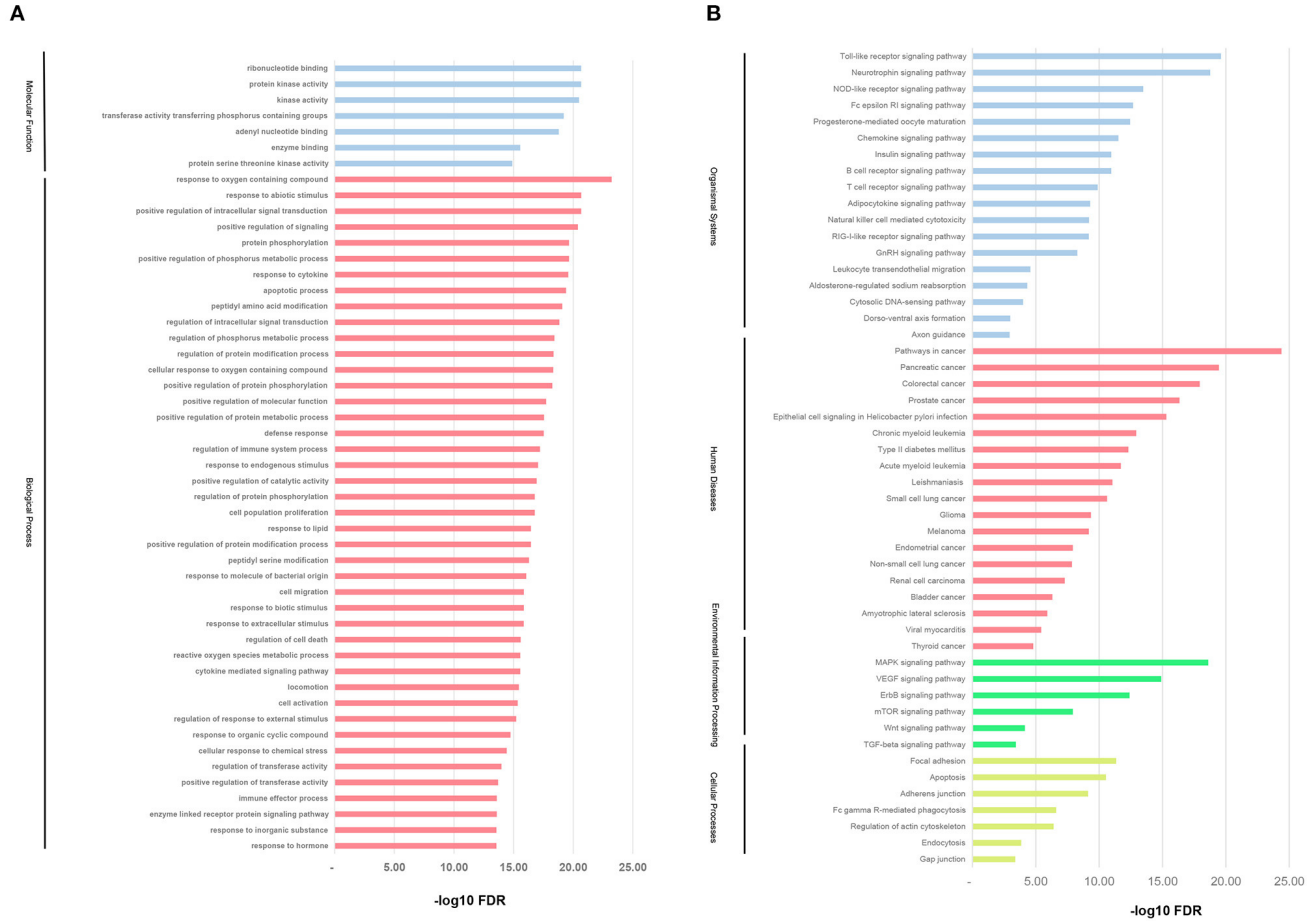
GO and KEGG pathway enrichment analysis. **(A)** GO analysis of the candidate direct targets of CR for KD. Functionally grouped network of enriched categories was generated for the target genes. **(B)** The KEGG pathway annotation of the candidate direct targets of CR for KD treatment.

To investigate the biological functions of the direct targets of CR for KD, the target genes GO and KEGG pathway function enrichment was performed by GSEA. As shown in Figure 4A, GO enrichment analysis showed that the top 50 GO terms were mainly biological processes with response to hormone, response to inorganic substance, enzyme-linked receptor protein signaling pathway, and immune effector process as the most significant (FDR <3.98E−21).

The KEGG pathway enrichment is shown in Figure 4B, mainly distributed in four major classes (Organismal Systems, Human Diseases, Cellular Processes, and Environmental Information Processing). The top five pathways were screened, namely, Pathways in cancer, Toll-like receptor signaling pathway, Pancreatic cancer, Neurotrophin signaling pathway, and MAPK signaling pathway.

**Figure 5:**
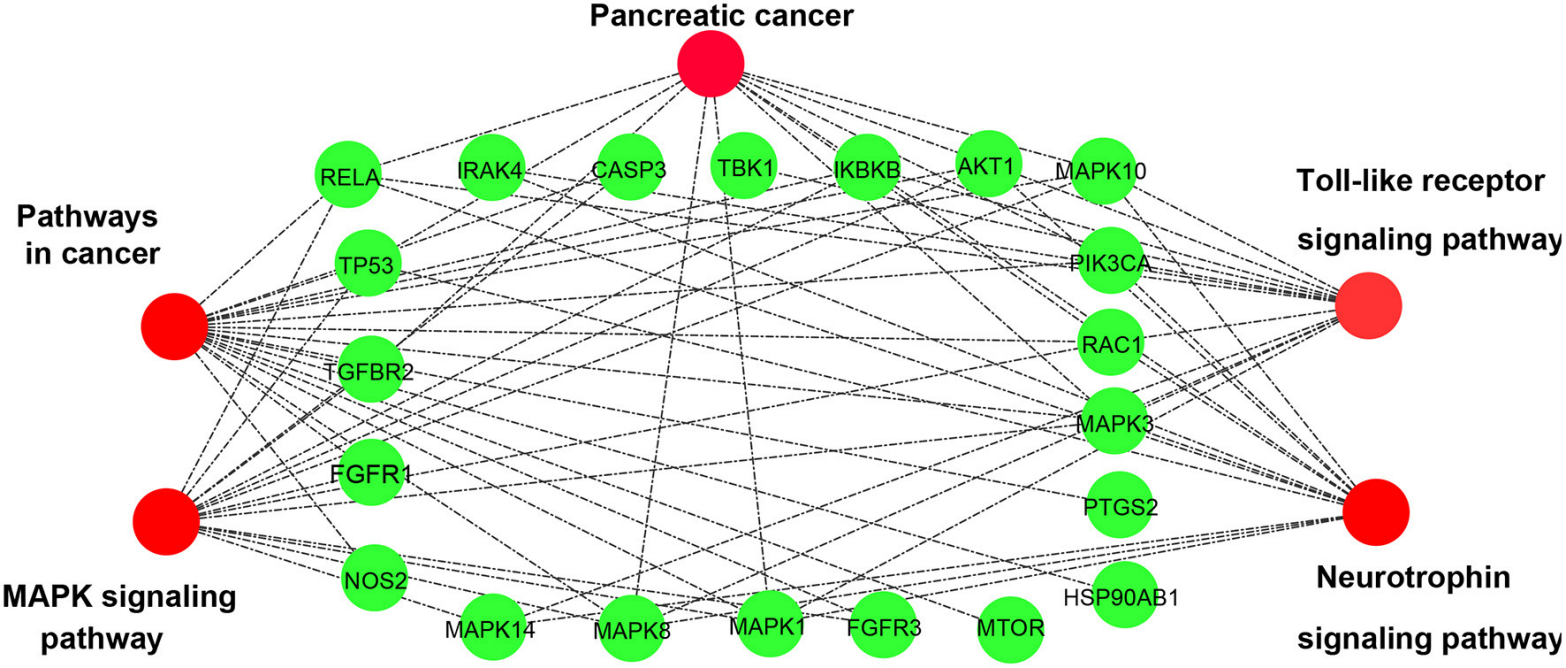
The signaling pathway diagram of the candidate direct targets of CR for KD treatment.

Cytoscape3.6.0 software was used to demonstrate the enrichment of the 21 proteins of the above five pathways (shown in Figure 5). Most of the genes are enriched in Pathways in cancer and MAPK signaling pathway, and these 12 genes of the latter are completely contained by the 18 genes of the former.

### Compound–Common Target Between Compound and KD Network Analysis

**Figure 6:**
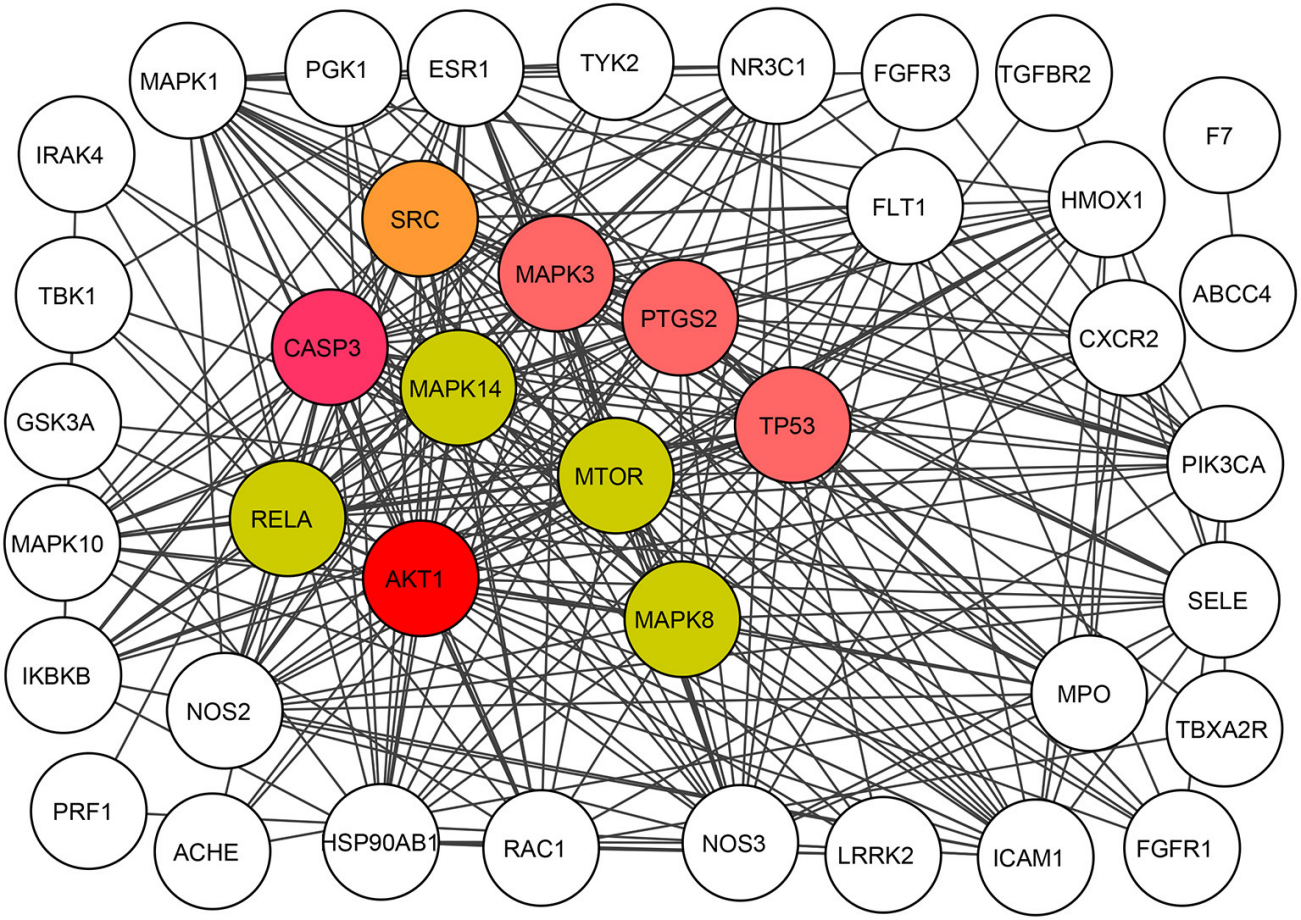
Protein interaction network of all compound-target genes encoding protein.

For this part of the study, the String online server was the main tool that helped to build the PPI network, containing 41 nodes in total (shown in Figure 6). The significant target proteins SRC, CASP3, MAPK3, PTGS2, TP53, AKT1, RELA, MAPK14, MTOR, and MAPK8 were screened according to the degree and colored. These genes were ranked in the top 10 (Table 1).

**Table 1:** Hub genes of CR for KD treatment.

**Gene names**	**Description**	**Degree**
*AKT1*	AKT serine/threonine kinase 1	30
*CASP3*	Caspase 3	28
*TP53*	Tumor protein p53	26
*MAPK3*	Mitogen-activated protein kinase 3	26
*PTGS2*	Prostaglandin-endoperoxide synthase 2	26
*SRC*	SRC proto-oncogene, non-receptor tyrosine kinase	25
*MAPK8*	Mitogen-activated protein kinase 8	23
*MAPK14*	Mitogen-activated protein kinase 14	23
*RELA*	RELA proto-oncogene, NF-kB subunit	23
*MTOR*	Mechanistic target of rapamycin	23

### Validation of the Protein Expression of PTGS2, CASP3, ERK, AKT1, and SRC After Berberine Treatment

**Figure 7:**
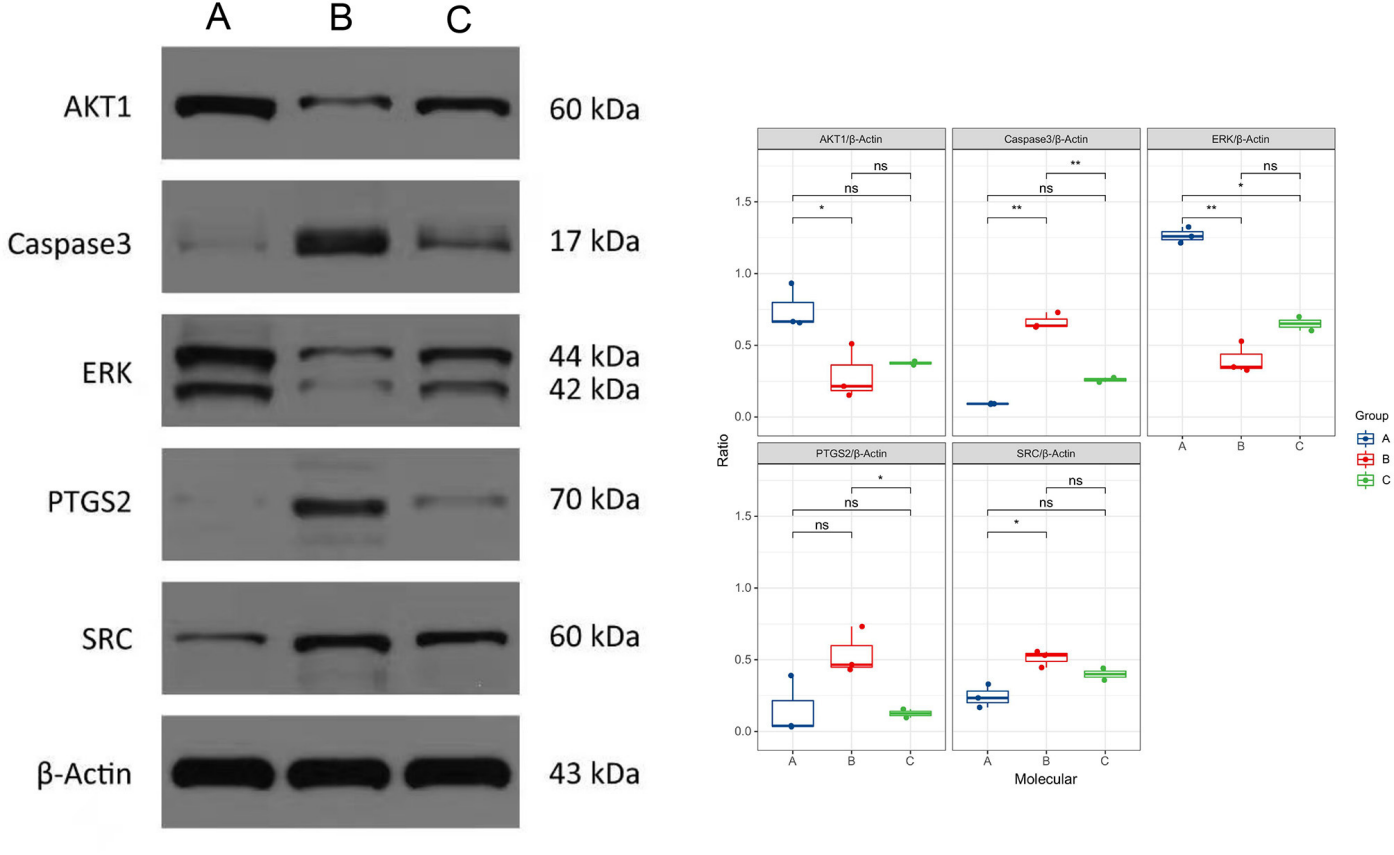
Expression of protein of five genes detected through Western blot. A means normal (*n* = 3), B means KD (*n* = 3), C means KD + Berberine (*n* = 2), ns means there was no statistically significant difference. Asterisk marks mean *p < 0.05 and **p < 0.01.

We selected the top 5 genes according to the degree of PPI, including PTGS2, CASP3, ERK, AKT1, and SRC. After the treatment of berberine, the expressions of CAPS3 and PTGS2 were extremely significantly decreased, and the difference was statistically significant. While the expression of SRC was decreased, the expression of ERK and AKT1 was increased, and there was no statistically significant difference (Figure 7).

## Discussion

KD is a systemic inflammatory syndrome of small and medium vessels. In recent years, more and more evidence has shown that the pathogenesis of this condition is related to infectious factors, susceptibility genes and autoimmune activation. Traditional Chinese medicine is suitable for treating diseases with complex mechanisms because of its multiple curative effects and minimal side effects. The effects of BBR on KD in clinical practice were tested based on some previous findings regarding this topic (13). The present study can be considered the first clinical trial to evaluate the effect of BBR in patients with KD. In the study, the network pharmacology method was used to analyze the possible molecular mechanisms of CR in the treatment of KD.

In the clinical trial stage, C-reactive protein (CRP) and peripheral blood cell parameters were also used as markers of systemic inflammation. Among them, CRP plays a role in promoting phagocytosis and immune regulation. Data have shown that during the course of KD, the increase of CRP level is related to coronary artery dilatation and is a high-risk factor of complication with coronary artery injury for KD (25). NLR and PLR are markers of the balance between inflammatory response and immune regulation and are associated with cardiovascular adverse events. Studies have shown that NLR is directly proportional to the intensity of the inflammatory response (26). At the same time, Turkmen et al. indicated that PLR might be more effective than NLR in predicting the severity of systemic inflammatory response (27). The present study indicated that the BBR treatment accelerates the reduction of CRP, NLR, and PLR, which means that BBR can alleviate the inflammatory response in patients with KD. The rate of IVIG resistance in the BBR treatment group was significantly lower than that of the control group, indicating that BBR increases the therapeutic efficiency of routine therapy in KD.

To investigate the mechanisms of BBR on KD, network pharmacology was used. Nine target compounds of CR were collected from TCMSP; 369 target genes of CR were collected from TCMSP, SWISS, SEA, and STITCH database; and a total of 624 target genes of CR were obtained by searching related databases together with 41 target genes obtained by the intersection of CR and KD. By further screening this sample of genes, *AKT1, CASP3, TP53, MAPK3/8/14, PTGS2, SRC, RELA*, and *MTOR* were found and set as top 10 target genes as well.

AKT1, a serine/threonine protein kinase, is widely expressed in various tissues. It is known that activated AKT plays a regulatory role in the cell cycle, apoptosis, and proliferation by activating downstream factors. Also, the specific activation of AKT1 in vascular endothelial cells can alleviate the injury after carotid artery ligation by increasing the expression of nitric oxide and protecting the function of the endovascular cortex (28). By initializing AKT1 in vascular smooth muscle cells, it is possible to effectively inhibit the apoptosis and negative remodeling of vascular smooth muscle cells after carotid artery ligation, highlighting the protective role of AKT1 in vascular remodeling (29). In addition, the study has confirmed that p21 phosphorylated by AKT1 in endothelial cells may promote angiogenesis and metastasis, suggesting that p21 phosphorylation may play an important role in KD coronary artery abnormalities (30).

MAPK, an intracellular serine/threonine protein kinase, is an important signaling system for cell-mediated extracellular signals to intracellular responses and plays a key role in cell proliferation, apoptosis, inflammation, immunity, and angiogenesis (31). Studies have found that inhibiting MAPK signaling pathway activation can reduce the occurrence of the inflammatory response (32). So, this research postulated that MAPK might play an important regulatory role in the occurrence and development of vasculitis in KD.

SRC encodes tyrosine protein kinase also has to be mentioned since it is a member of the non-receptor protein tyrosine kinase family. This protein is constantly associated with multiple signaling pathways in cells and its related genes are involved in important biological processes such as growth, differentiation, adhesion, and transcription. Studies have confirmed that the SRC-1 gene is related to the susceptibility to coronary artery aneurysm of KD complications (33), indicating that the SRC gene is linked to the regulatory mechanism of KD.

Additionally, CASP3 is a key apoptotic protease in the final pathway of apoptotic cell death, mediating exogenous and endogenous cell death signaling pathways. CASP3 leads to transcriptional activation of inflammatory genes by activating the NF-κB pathway in the mechanism of KD. As a genetic variation of genes may also cause damage and remodeling of vascular structures, TP53 is considered to be an important tumor suppressor gene that can affect cell cycle, DNA repair, apoptosis, signal transduction, transcription, and autophagy, as well as regulate the growth, differentiation, and senescence of cells. PTGS2 contains 10 exons and 9 introns, encoding cyclooxygenase 2 (Cox-2). Cox-2, in this case, is expressed in vascular smooth muscle, monocytes, and fibroblasts, and is a cardinal inflammatory mediator in the process of atherosclerosis (34). In addition, overexpression of COX-2 may cause inflammation of the vascular wall, plaque instability, and intimal hyperplasia (35). It is believed that the occurrence of this vascular wall inflammation is closely related to the mechanism of KD, and this hypothesis is consistent with the results of the network pharmacology processes here conducted.

RELA is a member of the NF-κB family. NF-κB plays a key role in inflammatory and immune responses in cells. Studies have shown that in the pathological process of KD, the NF-κB signaling system regulates transcription of almost all genes involved in inflammatory mediators and cell proliferation and activation. Activation of the NF-κB signaling pathway is linked to the occurrence of KD vasculitis in the acute phase, which is likely to aggravate KD vasculitis response and participate in the formation of coronary artery injury (36). ICAM-1 is considered an initiator of inflammatory cell adhesion and is also closely related to endothelial dysfunction (37), playing an important role in the development of cardiovascular disease (38, 39). After the endothelial injury, ICAM-1 expression increases, aggravating vascular injury by releasing more cytokines and chemokines (40). These findings are consistent with the network-pharmacologic outcomes of this study and these target genes could be potential candidates for KD.

In addition, the GO enrichment analysis in this study showed that response to hormone, response to inorganic substance, enzyme-linked receptor protein signaling pathway, and immune effector process are the major biological processes for CR treatment of KD. The disturbance of these biological processes is likely the cause of KD vasculitis. Accordingly, CR may be important in the regulation of different biological functions of KD.

Finally, the enrichment analysis of the KEGG pathway found that the five signaling pathways closely related to CR and KD prevention and treatment included Pathways in cancer, Toll-like receptor signaling pathway, Pancreatic cancer, Neurotrophin signaling pathway, and MAPK signaling pathway. The Toll-like receptor signaling pathway is a family of receptors composed of members of multiple receptors and has a key function when it comes to inflammatory and immune injuries of endothelial cells caused by pathogen infection and immune response (41). Clinical studies have been helpful to confirm the correlation between the TLR signaling pathway and inflammatory immune injuries of vascular endothelial cells in KD (42). Furthermore, the present results suggested that KD serum-associated miR-186 has an essential role in endothelial cell apoptosis by activating the MAPK pathway through targeting the SMAD6 gene (43). Thus, we may conclude the key role of MAPK pathway in the pathogenesis of KD. Also, the study may help to reveal the potential targets for novel therapy of KD.

This study systematically explored the putative bioactive compounds in CR and pharmacological targets of CR for KD prevention and treatment through network pharmacology and Western blotting. All the ingredients of CR were extracted from edible plants, and they were all claimed safe in previous applications. Therefore, this study provides a new way to explore the mechanism of CR in treating KD. However, the examinations here conducted have certain limitations. It is suggested for the future that relevant experiments are carried out to better explain the mechanisms of CR in the treatment of KD.

## Conclusion

After conducting this examination, we postulated that BBR is effective in the treatment of young children with KD and can be considered as an alternative treatment for KD. This study has preliminarily revealed the possible mechanism of CR in the treatment of this disease by regulating multi-targets with multi-components. Furthermore, the outcomes demonstrated that a network pharmacology-based approach was useful for elucidation of the interrelationship between complex diseases and interventions of Chinese herbal medicines, potentially providing references for further research on its mechanisms of action in the future.

## Data Availability Statement

The original contributions presented in the study are included in the article/Supplementary Materials, further inquiries can be directed to the corresponding author/s.

## Ethics Statement

The studies involving human participants were reviewed and approved by Shenzhen CHildren Hospital. Written informed consent to participate in this study was provided by the participants' legal guardian/next of kin.

## Author Contributions

MX conceived and designed the research, and reviewed and revised the manuscript. XF wrote the manuscript. XG and YL performed a preliminary analysis. All authors read and approved the final version of the manuscript.

## Funding

This study was supported by the National Nature Science Foundation of China (81870364), Shenzhen Sanming Project (SZSM20162057), Shenzhen Scientific Plan (JCYJ20190809164004023), and Guangdong Province High Level Clinical Key Specialties (SZGSP012) (all from MX).

## Conflict of Interest

The authors declare that the research was conducted in the absence of any commercial or financial relationships that could be construed as a potential conflict of interest.

## Publisher's Note

All claims expressed in this article are solely those of the authors and do not necessarily represent those of their affiliated organizations, or those of the publisher, the editors and the reviewers. Any product that may be evaluated in this article, or claim that may be made by its manufacturer, is not guaranteed or endorsed by the publisher.

## Abbreviations

CR: Coptidis Rhizoma
KD: Kawasaki disease
TCMSP: Traditional Chinese Medicine Systems Pharmacology
SEA: Similarity Ensemble Approach
GO: Gene Ontology
KEGG: Kyoto Encyclopedia of Genes and Genomes
CAA: coronary artery aneurysms
IVIG: intravenous gamma globulin
HL: Huang Lian
BBR: Berberine
DL: drug-likeness
OB: oral bioavailability
BBR: blood–brain barrier
PPI: protein–protein interaction
CRP: C-reactive protein
NLR: neutrophils/lymphocytes
PLR: platelets/lymphocytes.
